# Hand-Assisted Laparoscopic Donor Nephrectomy in Complete Situs Inversus

**DOI:** 10.1089/cren.2016.0045

**Published:** 2016-05-01

**Authors:** John V. Gahagan, Matthew D. Whealon, Uttam Reddy, Clarence E. Foster, Hirohito Ichii

**Affiliations:** ^1^ Department of Surgery, University of California, Irvine School of Medicine, Orange, California.; ^2^ Department of Medicine, University of California, Irvine School of Medicine, Orange, California.

## Abstract

Complete situs inversus is a rare congenital anomaly characterized by transposition of organs. We report a case of renal transplantation using a kidney from a living complete situs inversus donor. The recipient was a 59-year-old female with end-stage renal disease because of type 2 diabetes mellitus. The donor was the 56-year-old sister of the recipient with complete situs inversus. CT angiogram of the abdomen and pelvis showed complete situs inversus and an otherwise normal appearance of the bilateral kidneys with patent bilateral single renal arteries and longer renal vein in the right kidney. The patient was taken to the operating room for a hand-assisted laparoscopic right donor nephrectomy. The patient tolerated the procedure well and was discharged home in good condition on postoperative day 1. The recipient experienced no episodes of acute rejection or infection, with serum creatinine levels of 0.8–1.2 mg/dL. Laparoscopic donor nephrectomy in a patient with complete situs inversus remains a technically feasible operation and the presence of situs inversus should not preclude consideration for living kidney donation.

## Introduction and Background

Complete situs inversus is a rare syndrome which has laterality reversal of thoracic and abdominal organs. It affects ∼1 in 6000–8000 individuals and the etiology is not completely understood.^1^ The anatomic relationship between organs is unchanged, except that the configuration is a mirror image of the typical anatomic position. This reversal can give pause or confusion to physicians and surgeons performing an examination or a procedure. We present a contemporary case report of a hand-assisted laparoscopic donor nephrectomy in a patient with complete situs inversus.

## Presentation of Case

**FIG. 1.**
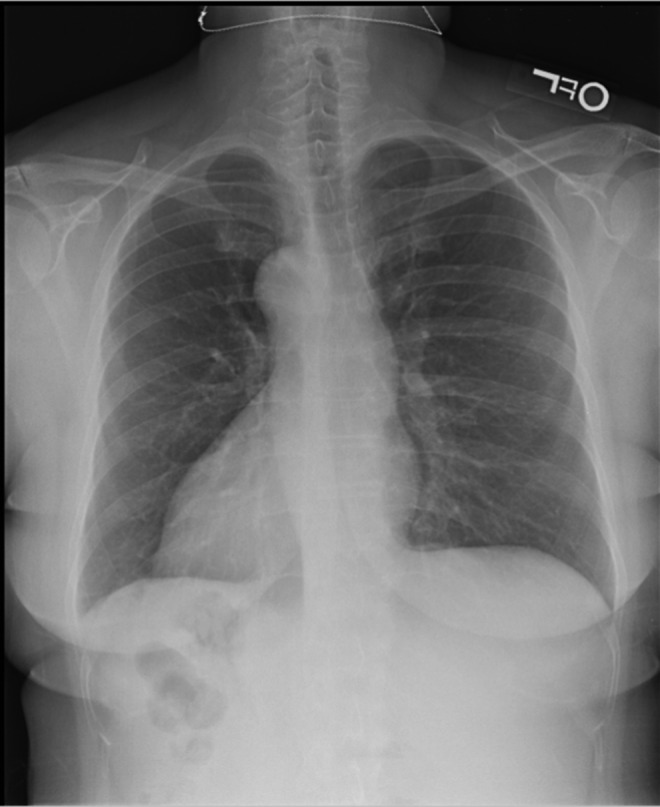
Chest radiograph shows dextrocardia.

**FIG. 2.**
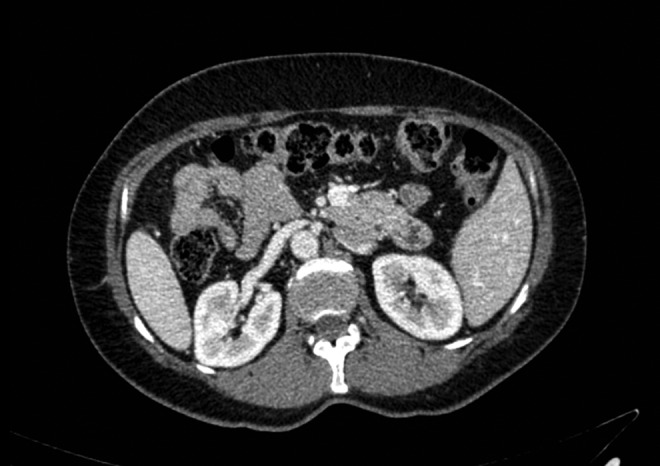
Preoperative donor CT angiogram shows longer right renal vein.

The donor is a 56-year-old female with complete situs inversus who volunteered to donate a kidney to her sister. The recipient is a 59-year-old female with end-stage renal disease because of type 2 diabetes mellitus. The recipient has a history of hypertension for which she takes amlodipine. Her surgical history includes an open appendectomy and cesarean section. Laboratory values during preoperative planning were unremarkable, with a creatinine of 0.7 mg/dL. Her BMI was 25.4 and her physical examination was otherwise normal in appearance. CT angiogram of the abdomen and pelvis showed situs inversus and an otherwise normal appearance of the bilateral kidneys with patent bilateral single renal arteries (Fig. 1 and 2). Echocardiography was unremarkable with a normal left ventricular ejection fraction. Mammography was normal.

**FIG. 3.**
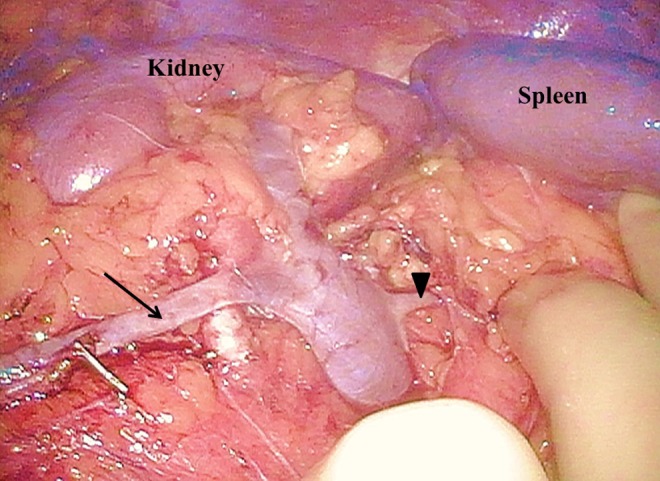
The right gonadal vein (*arrow*) and right adrenal vein (*arrowhead*) of the donor were bifurcated from right renal vein.

**FIG. 4.**
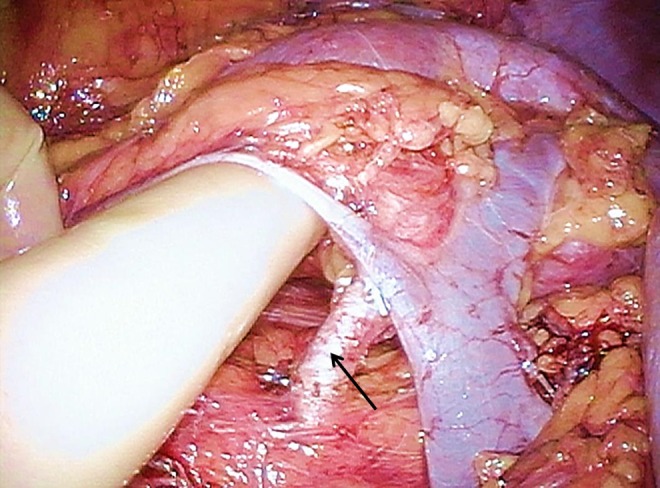
Right renal artery (*arrow*) of the donor was identified behind renal vein.

The patient was taken to the operating room for a hand-assisted laparoscopic right donor nephrectomy. General anesthesia with orotracheal intubation was initiated and a Foley catheter was inserted. She was placed in the left lateral decubitus position with the bed flexed. A 7 cm vertical incision was made at the umbilicus through which we placed a wound protector and a GelPort (Applied Medical, Rancho Santa Margarita, CA). With a 5 mm trocar in the GelPort, the abdomen was insufflated to 15 mmHg pressure. The patient was then given 12.5 g of mannitol. Two 5 mm trocars were placed: 3 cm (working) and 7 cm (camera) caudal from the right costal margin at the lateral border of the rectus abdominus. The left hand was inserted to the abdomen through the GelPort. After lysis of adhesions, the descending colon was mobilized along the white line of Toldt to the iliac vessels. The mesentery of the colon was dissected off the kidney, and Gerota's fascia was opened. We identified the gonadal vein and ureter, which were dissected together with fat and connective tissue. The ligamentous attachment between the spleen and the upper pole of the kidney was identified and dissected. The right adrenal vein was identified at the medial side (Fig. 3). Since the length of right renal vein was enough for transplant, we elected to preserve the adrenal vein. The adrenal gland was identified and dissected from the kidney. The renal artery was located at the distal and posterior side of the right renal vein (Fig. 4). Once the kidney had been completely dissected free, and the renal artery and vein had been both isolated, heparin 5000 units and mannitol 12.5 g were given intravenously. The lower 5 mm trocar was upsized to a 12 mm trocar to accommodate a laparoscopic stapler. After 3 minutes, we divided the distal ureter and the gonadal vein with vascular stapler (ENDOPATH^®^ ETS Articulating Linear Cutters, Ethicon). We then sequentially divided the renal artery and renal vein, each with the vascular stapler. The kidney was extracted through the midline incision. The warm ischemia time was less than 1 minute. The cold ischemia time was 85 minutes. The staple lines of both renal artery and vein were observed and were without evidence of bleeding. All incisions were closed. The patient tolerated the procedure well and was discharged home in good condition on postoperative day 1.

## Discussion and Literature Review

The technique of laparoscopic donor nephrectomy was first described in 1995 by Ratner et al.^2^ Since that time, the laparoscopic approach to a donor nephrectomy has been widely accepted and recognized as a safe procedure.^3,4^ In a majority of the laparoscopic donor nephrectomies, the left kidney is removed because of the technical ease of a longer vein and the absence of the liver and duodenum on that side. In the case of situs inverus, this anatomy is reversed, such that the patient's right kidney has a longer vein and the liver and duodenum are far away. In planning this case, we anticipated that we would place our incisions as though we were performing a standard right nephrectomy. After observing the right upper quadrant, this positioning seemed appropriate, although the anatomy was initially quite unique. The preoperative CT was crucial in our ability to anticipate the anatomy.

Laparoscopic appendectomy, cholecystectomy, and colectomy, among other procedures, in patients with compete situs inversus have previously been reported. The key conclusion in many of these reports is the importance of preoperative planning and understanding of the anatomy. This foresight allows surgeons to alter the operative technique before the operation and reorient their mind to the mirrored anatomy.

## Conclusion

From our experience with this patient, laparoscopic donor nephrectomy in a patient with complete situs inversus remains a technically feasible operation and the presence of situs inversus should not preclude consideration for kidney donation.

## Abbreviations Used

CT: computed tomography
